# B.T.C.H. nephrolithometry score: a novel scoring system to predict stone-free rate and complexity for ultrasound-guided percutaneous nephrolithotomy

**DOI:** 10.3389/fmed.2025.1557702

**Published:** 2025-05-08

**Authors:** Haiwen Huang, Haifeng Song, Tianfu Ding, YangYang Xu, Yunfei Fan, Boxing Su, Yubao Liu, Weiguo Hu, Bo Xiao, Jianxing Li

**Affiliations:** Department of Urology, Beijing Tsinghua Changgung Hospital, School of Clinical Medicine, Tsinghua Medicine, Tsinghua University, Beijing, China

**Keywords:** percutaneous nephrolithotomy, kidney stone, stone-free rate, CT urography, scoring system

## Abstract

**Background:**

The existing scoring systems for percutaneous nephrolithotomy fail to adequately consider the influence of renal anatomy, leading to limited predictive accuracy. This study introduces and validates a novel B.T.C.H. nephrolithometry score, designed to better predict stone-free rates and complexity for ultrasound-guided percutaneous nephrolithotomy.

**Methods:**

B.T.C.H. nephrolithometry score evaluates four variables including stone burden, type of renal pelvis, calyces involved, and hydronephrosis. 134 patients who underwent ultrasound-guided percutaneous nephrolithotomy at Beijing Tsinghua Changgung Hospital were retrospectively analyzed. The inter-observer agreement was assessed using the linearly weighted kappa coefficient. The accuracy in predicting the stone-free rate was evaluated using receiver operating characteristic curve analysis. Spearman’s correlation analysis and Kendall’s W test were employed to examine the correlation between the scores of each scoring system and operative time, the number of tracts and CDC scores.

**Results:**

The overall stone-free rate was 52.99%. The stone-free rates in low (4–8 points), medium (9–12 points), and high (13–15 points) B.T.C.H. scores were 91.9, 24.6, and 0%, respectively. The B.T.C.H. nephrolithometry score had an AUC of 0.909 for predicting stone-free rate, outperforming both the GSS (AUC = 0.761) and the S.T.O.N.E. nephrolithometry score (AUC = 0.763). The B.T.C.H. nephrolithometry score were positively correlated with operative time, the number of tracts and CDC scores.

**Conclusion:**

B.T.C.H. nephrolithometry score is a suggested novel scoring system for ultrasound-guided percutaneous nephrolithotomy, which had superior prediction of stone-free rate and positive correlation with operative time, the number of tracts, and postoperative CDC scores.

## 1 Introduction

Percutaneous nephrolithotomy (PCNL) is widely recognized as the standard treatment for renal calculi, particularly in complex cases (1). However, due to the significant variability in the characteristics of renal stones across different cases, the complexity of PCNL procedures and the postoperative stone-free rate (SFR) vary considerably. To address this variability, numerous stone scoring systems have been developed to classify cases and predict outcomes, such as Guy’s stone score (2), S.T.O.N.E. nephrolithometry (3), the CROES nomogram (4), and S-ReSC (5). These scoring systems have been proven to correlate with the SFR and the incidence of complications (6). However, these scoring systems were developed based on X-ray-guided PCNL and did not adequately consider the anatomical variations of the renal pelvis that can influence surgical difficulty, leading to a less significant correlation between these scoring systems and the postoperative prognosis and surgical complexity in ultrasound-guided PCNL. Furthermore, some scoring systems are complex, lacking quantifiable indicators, which limits their usability and clinical applicability.

Present study proposes a novel scoring system, the B.T.C.H. nephrolithometry score, designed for ultrasound-guided PCNL. This scoring system not only assesses key factors such as stone burden, stone distribution, and hydronephrosis, but also integrates anatomical characteristics of the renal pelvis. We evaluated the consistency of this scoring system and retrospectively reviewed PCNL cases at our center to validate the predictive performance of the scoring system and evaluate its correlation with the difficulty of PCNL procedures.

## 2 Materials and methods

### 2.1 B.T.C.H. nephrolithometry score

The B.T.C.H. nephrolithometry score is a novel scoring system based on preoperative CT urography (CTU) imaging. Unlike other scoring systems that rely on non-contrast CT imaging, CTU provides comprehensive information not only about the kidney stones but also about the anatomical structure of renal pelvicalyceal system. The B.T.C.H. nephrolithometry score evaluates four key variables, which are abbreviated using the acronym “B.T.C.H.” The four variables include stone burden (“B”), type of renal pelvis (“T”), calyces involved (“C”), and hydronephrosis (“H”).

**Figure 1:**
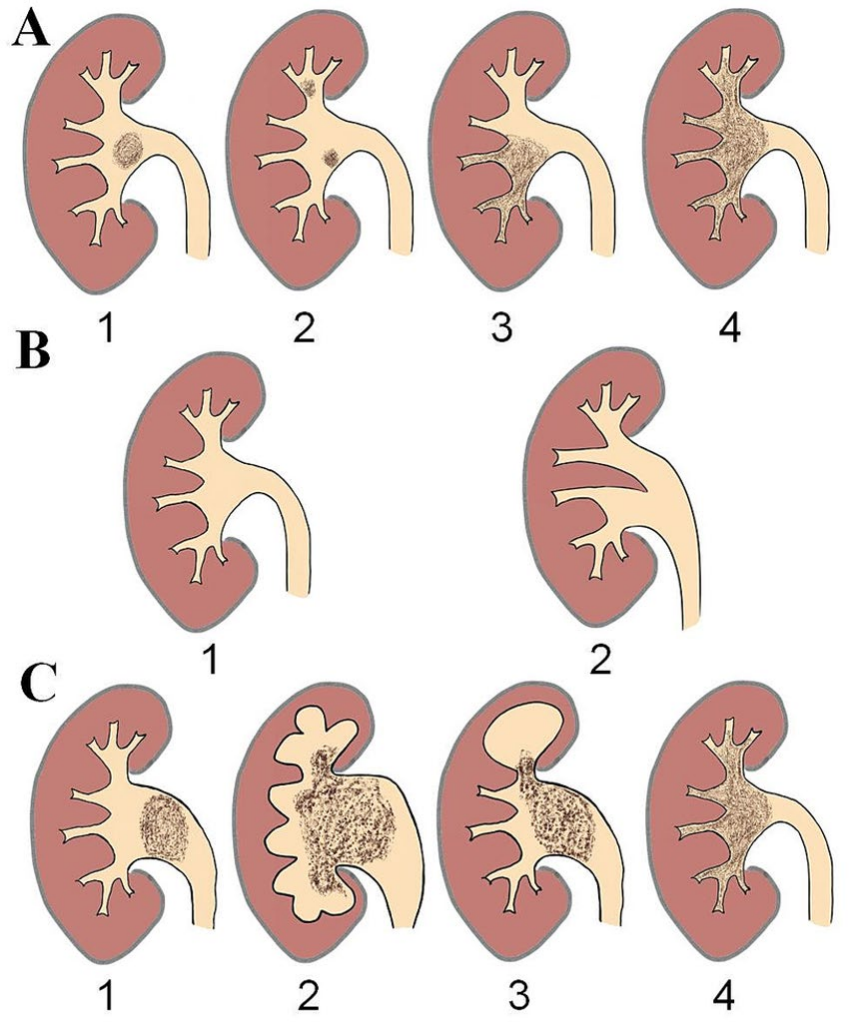
Scores for different B.T.C.H. variables. **(A)** Stone burden, 1: solitary stone, 2: multiple stone, 3: partial staghorn stone, 4: complete staghorn stone. **(B)** Type of pelvis, 1: single pelvis, 2: divided pelvis. **(C)** Hydronephrosis. 1: mild hydronephrosis, 2: severe hydronephrosis, 3: localized hydronephrosis caused by obstruction of the renal calyx infundibulum, 4: no hydronephrosis.

The “stone burden” is evaluated based on the amount and the size of the kidney stone in the patients, with higher scores indicating a greater stone burden (Figure 1A). A score of 1 point is assigned for solitary stone, while the case with multiple renal stones is given a score of 2 points. For the partial staghorn stones, the score is 3 points, and for complete staghorn stones, the score is 4 points.

Based on the CTU urography reconstructions, the pelvis is categorized into two types according to the branch patterns: single pelvis and divided pelvis (7). For scoring of the “type of pelvis,” single pelvis is assigned a score of 1 point, while divided pelvis is assigned a score of 3 points (Figure 1B).

The “calyces involved” is scored based on the number of calyces affected by kidney stones. If 1–2 calyces are involved, a score of 1 point is assigned. In cases of 3–4 calyces are involved, the score is 2 points. When 5 or more calyces are affected, a score of 3 points is given. If the number of involved calyces cannot be evaluated due to anatomical abnormalities of the kidney, a score of 4 points is assigned.

The last scoring item, “hydronephrosis,” is scored based on the severity of hydronephrosis (Figure 1C). Mild hydronephrosis is assigned a score of 1 point, while severe hydronephrosis is scored as 2 points. If localized hydronephrosis caused by obstruction of the renal calyx infundibulum, a score of 3 points is given. And cases without hydronephrosis are assigned the highest score of 4 points.

The scoring criteria of the B.T.C.H. nephrolithometry score are detailed in Table 1. The total score is calculated by summing the scores of the four individual components, with a minimum score of 4 points and a maximum score of 15 points. A total score of 4–8 points indicates low complexity, 9–12 points represents moderate complexity, and 13–15 points corresponds to high complexity.

**Table 1:** B.T.C.H. nephrolithometry score.

Variable	Score			
	1	2	3	4
B, stone burden	Solitary stone	Multiple stone	Partial staghorn stone	Complete staghorn stone
T, type of pelvis	Single pelvis	–	Divided pelvis	–
C, calyces involved	1–2	3–4	≥5	Unevaluable due to abnormal anatomy
H, hydronephrosis	Mild	Severe	Localized hydronephrosis	No hydronephrosis

### 2.2 Patients and perioperative data

Present study retrospectively analyzed patients who underwent ultrasound-guided PCNL at Beijing Tsinghua Changgung Hospital in 2018. The inclusion criteria were: (1) age ≥18 years, and (2) undergoing PCNL at Beijing Tsinghua Changgung Hospital between January 2018 and January 2019. The exclusion criteria were: (1) concurrent ureteral or bladder stones, (2) missing perioperative imaging data, (3) undergoing a second-stage PCNL, (4) undergoing bilateral procedures during the same operation, (5) preoperative placement of a nephrostomy tube or ureteral stent, and (6) congenital renal anatomical abnormalities. Finally, a total of 134 patients were included for analyzing. This study collected clinical characteristics including age, gender, the Body Mass Index (BMI), the American Society of Anesthesiologists (ASA) score, operative time, and the number of tracts. Postoperative complications were graded using the Clavien-Dindo Classification (CDC) (8). Each patient’s stone complexity was assessed using the Guy’s stone score (2), S.T.O.N.E. nephrolithometry score (3), and B.T.C.H. nephrolithometry score. Additionally, for consistency testing, all cases were independently evaluated using the B.T.C.H. scoring system by another senior attending urologist. The urologists were blinded to the SFR and the perioperative data of patients when scored. On the second postoperative day, patients all underwent a kidney–ureter-bladder (KUB) X-ray. If no residual stones were observed, a follow-up CT scan was performed 1 month postoperatively to confirm the final stone-free status. If residual stones were detected on the postoperative KUB, patients were managed according to clinical need, including either a second-stage procedure or observation. Patients without residual stones or stone fragments ≤4 mm were classified as stone-free status. Ethical approval for this study was obtained from the clinical research ethics committee of Beijing Tsinghua Changgung Hospital, Beijing, China.

### 2.3 Surgical technique

The technique of ultrasound-guided PCNL was descripted previously (9, 10). Firstly, with the patient in the lithotomy position, a retrograde 5-Fr ureteral catheter was inserted into the renal pelvis to induce artificial hydronephrosis. Renal access was subsequently obtained under ultrasound guidance (using a 3.5-MHz probe, LOGIQ e; GE Healthcare, Wauwatosa, WI, USA) with the patient in the prone position, utilizing a 17.5-G needle. The tract was dilated to 24-Fr using either Amplatz sequential dilators or a high-pressure balloon dilator (X Force N30 balloon dilator; Bard Urological, Covington, GA, USA). Stone fragmentation was achieved using a combined ultrasonic and pneumatic lithotripter (Swiss LithoClast; EMS Electro Medical Systems, Nyon, Switzerland). At the end of the procedure, a 6-Fr double-J stent was placed antegrade into the ureter, and 14-Fr nephrostomy tubes were inserted for every tract.

### 2.4 Statistical analysis

First, the inter-observer agreement of the B.T.C.H. nephrolithometry score was assessed using the linearly weighted kappa coefficient. Then, the scores of each component and the total scores of the B.T.C.H. nephrolithometry score were compared between stone-free and non-stone-free patients using either the Chi-square test or Mann–Whitney U test. The accuracy of the B.T.C.H. nephrolithometry score and other scoring systems in predicting the SFR was evaluated using receiver operating characteristic (ROC) curve analysis. Spearman’s correlation analysis was employed to examine the correlation between the scores of each scoring system and operative time, while Kendall’s W test was used to analyze the correlation between the scores of each scoring system and the number of tracts or CDC scores. The statistical analyses were performed using IBM SPSS version 25 (IBM Corporation, Armonk, NY, USA), and all *p* values were two sided. *p* < 0.05 was considered to be statistically significant.

## 3 Results

A total of 134 patients who received ultrasound-guided PCNL were enrolled in present study. The clinical characteristics and perioperative data were summarized in Table 2. Of the 134 patients, 71 patients achieved stone-free status postoperatively, resulting in an overall stone-free rate (SFR) of 52.99%. The median operative time was 109 (74–133) minutes. Postoperative complications occurred in 47 patients, with major complications (CDC grade ≥3) observed in 4 patients. The median B.T.C.H. score was 9 (6–12) points. The weighted kappa for inter-observer agreement were 0.835 (95% confidence interval [CI] 0.706–0.963), indicating that the B.T.C.H. nephrolithometry score could be interpreted as having “very good reliability.”

**Table 2:** Clinical characteristics of study cohort.

Variables	Value
No. patients	134
Age (years)	53 (43–59)
Gender	
Male	80 (59.70%)
Female	54 (40.30%)
BMI	25.50 ± 3.62
ASA scores	
1	13 (9.70%)
2	117 (87.31%)
3	4 (2.99%)
CDC	
0	87 (64.93%)
1	23 (17.16%)
2	19 (14.18%)
3	5 (3.73%)
Operation Time (min)	109 (74–133)
Guy’s stone score	
Grade I	13 (9.70%)
Grade II	27 (20.15%)
Grade III	61 (45.52%)
Grade IV	33 (24.63%)
S.T.O.N.E. nephrolithometry score	
Low	69 (51.49%)
Medium	60 (44.78%)
High	5 (3.73%)
B.T.C.H. score	9 (6–12)
Stone-free	71 (52.99%)

BMI, Body Mass Index; ASA, American Society of Anesthesiologists; CDC, Clavien-Dindo class.

### 3.1 Stone-free status

The results of stone-free status and the SFR for patients across different B.T.C.H. score are detailed in Table 3, showing a progressive decline in SFR with increasing B.T.C.H. score. Patients were categorized into three groups based on their B.T.C.H. scores: the low-complexity group (4–8 points) with an SFR of 91.90%, the medium-complexity group (9–12 points) with an SFR of 24.6%, and the high-complexity group (13–15 points) with an SFR of 0%.

**Table 3:** SFR of patients with different score of B.T.C.H.

Items	*N*	No. stone-free	SFR
B.T.C.H. score			
4	11	11	100.00%
5	22	22	100.00%
6	10	9	90.00%
7	8	7	87.50%
8	11	8	72.70%
9	9	2	22.20%
10	13	5	38.50%
11	14	2	14.30%
12	21	5	23.80%
13	7	0	0.00%
14	7	0	0.00%
B.T.C.H. group			
Low (4–8)	62	57	91.90%
Medium (9–12)	57	14	24.60%
High (13–15)	14	0	0.00%
Total	134	71	52.99%

SFR, stone free rate.

We compared the total B.T.C.H. scores and each component scores between stone-free group and non-stone-free group, as shown in Table 4. The median B.T.C.H. score in the stone-free group was 6 (5–8) points, while in the non-stone-free group it was 11 (10–12) points, showing a significantly higher score in the non-stone-free group (*p*<0.001). Additionally, each component score of the B.T.C.H. nephrolithometry score was also significantly higher in the non-stone-free group compared to the stone-free group.

**Table 4:** Results of B.T.C.H. scores for stone-free patients and non stone-free patients.

Items	Stone-free	Non stone-free	*p*
B, Stone burden			<0.001
1	19 (26.8%)	0	
2	35 (49.3%)	21 (33.3%)	
3	10 (14.1%)	16 (25.4%)	
4	7 (9.9%)	26 (41.3%)	
T, Type of pelvis			<0.001
1	66 (93.0%)	42 (66.7%)	
3	5 (7.0%)	21 (33.3%)	
C, Calyces involved			<0.001
1	44 (62.0%)	2 (3.2%)	
2	10 (14.1%)	3 (4.8%)	
3	16 (22.5%)	30 (47.6%)	
4	1 (1.4%)	28 (44.4%)	
H, Hydronephrosis			<0.001
1	42 (59.2%)	4 (6.3%)	
2	11 (15.5%)	3 (4.8%)	
3	9 (12.7%)	41 (65.1%)	
4	9 (12.7%)	15 (23.8%)	
B.T.C.H.	6 (5–8)	11 (10–12)	<0.001

**Figure 2:**
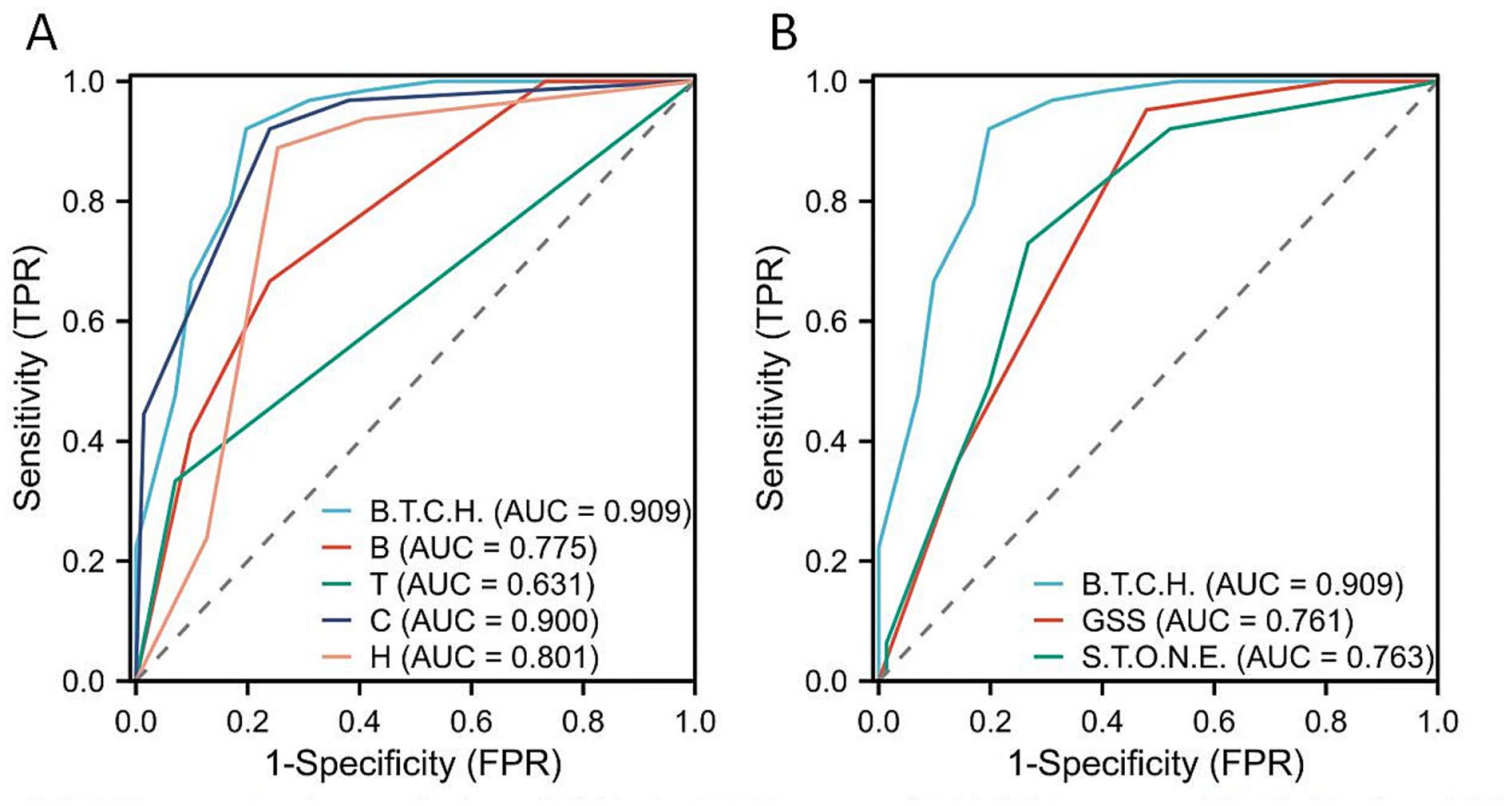
ROC curve for the prediction of SFR. **(A)** ROC curve of B.T.C.H. score and individual variables. **(B)** ROC curve of B.T.C.H. score, Guy’s stone score and S.T.O.N.E. nephrolithometry score.

ROC curve analysis was performed to evaluate the accuracy of the B.T.C.H. nephrolithometry score and other scoring systems in predicting SFR. As shown in Figure 2, the B.T.C.H. nephrolithometry score had an AUC of 0.909 for predicting SFR, outperforming both the GSS (AUC = 0.761) and the S.T.O.N.E. nephrolithometry score (AUC = 0.763).

### 3.2 Perioperative data

**Figure 3:**
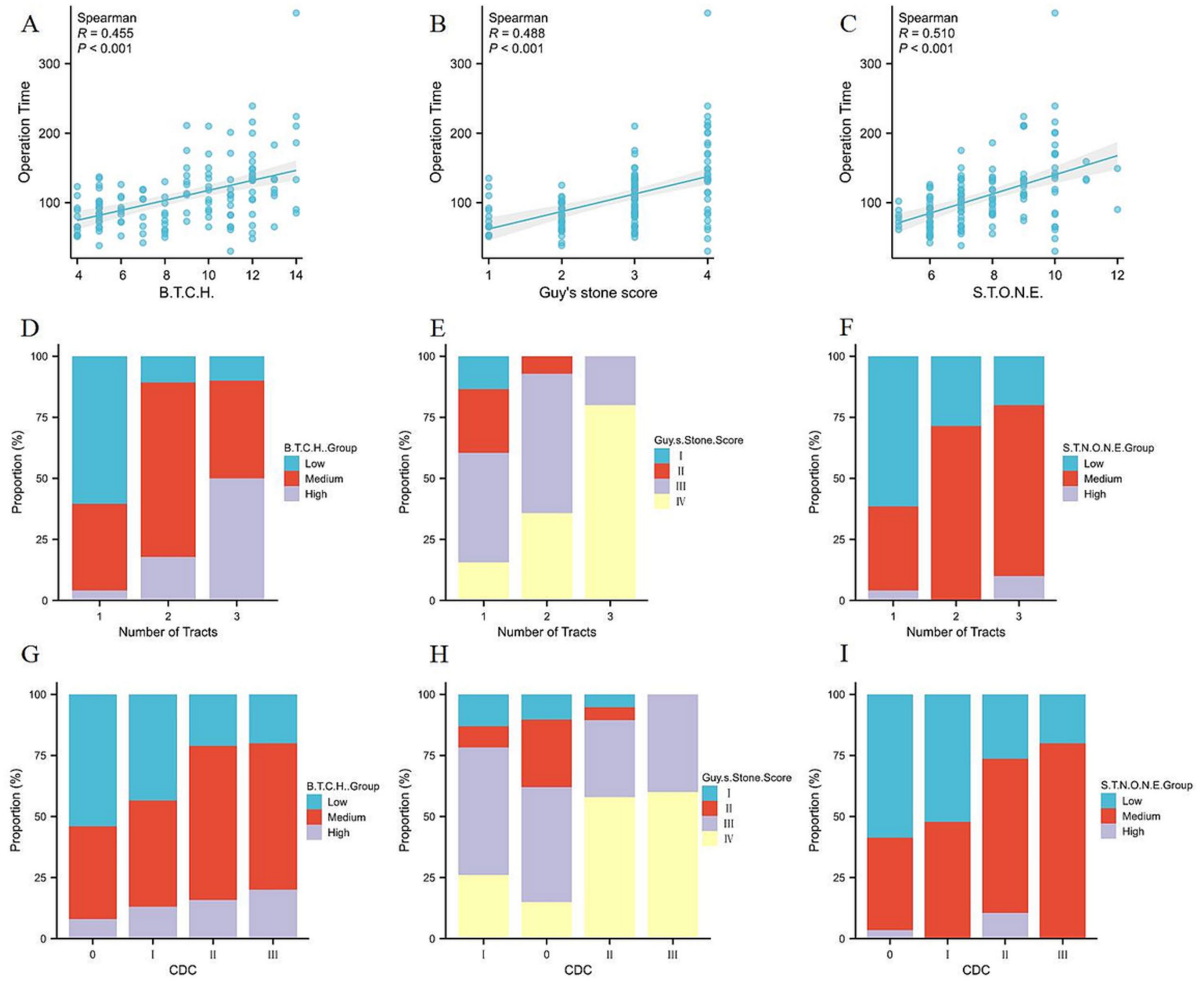
Correlation between different scoring system vs. operation time, number of tracts or CDC. **(A)** Correlation between B.T.C.H. score and operation time, *R* = 0.455, *p*<0.001. **(B)** Correlation between Guy’s stone score and operation time, *R* = 0.488, *p*<0.001. **(C)** Correlation between S.T.O.N.E. score and operation time, *R* = 0.510, *p*<0.001. **(D)** Correlation between B.T.C.H. group and number of tracts, Kendall’s tau-*b* = 0.471, *p*<0.001. **(E)** Correlation between Guy’s stone score and number of tracts, Kendall’s tau-*b* = 0.389, *p*<0.001. **(F)** Correlation between S.T.O.N.E. group and number of tracts, Kendall’s tau-*b* = 0.289, *p*<0.001. **(G)** Correlation between B.T.C.H. group and CDC, Kendall’s tau-*b* = 0.219, *p* = 0.006. **(H)** Correlation between Guy’s stone score and CDC, Kendall’s tau-*b* = 0.303, *p*<0.001. **(I)** Correlation between S.T.O.N.E. group and CDC, Kendall’s tau-*b* = 0.207, *p* = 0.006.

In terms of surgical complexity, we evaluated the correlation of different scoring systems with operative time and the number of tracts. All three scoring systems (B.T.C.H., GSS, and S.T.O.N.E.) were positively correlated with operative time and the number of tracts. The S.T.O.N.E. nephrolithometry score had a more accurate prediction of the operation time (*r* = 0.510, *p*<0.001 vs. *r* = 0.455, *p*<0.001 and *r* = 0.488, *p*<0.001), compared with B.T.C.H. nephrolithometry score and GSS (Figures 3A–C). While the B.T.C.H. nephrolithometry score showed a stronger correlation with the number of tracts (Kendall’s tau-*b* = 0.471, *p*<0.001 vs. Kendall’s tau-*b* = 0.389, *p*<0.001 and Kendall’s tau-*b* = 0.289, *p*<0.001) compared with GSS and S.T.O.N.E. nephrolithometry score (Figures 3D–F).

Regarding postoperative complications, the correlation between various scoring systems and postoperative CDC scores were also analyzed. Positive correlations were observed between the scores of the B.T.C.H. nephrolithometry score (Kendall’s tau-*b* = 0.219, *p* = 0.006), GSS (Kendall’s tau-*b* = 0.303, *p* < 0.001), and S.T.O.N.E. nephrolithometry score (Kendall’s tau-*b* = 0.207, *p* = 0.006) with the CDC scores, with GSS showing the strongest correlation (Figures 3G–I).

## 4 Discussion

In this study, we proposed a novel ultrasound-guided PCNL scoring system: the B.T.C.H. nephrolithometry score. This system evaluates four parameters based on preoperative CTU imaging: stone burden, type of pelvis, calyces involved, and hydronephrosis. The B.T.C.H. nephrolithometry score was validated using our center’s cohort, demonstrating effective prediction of SFR following PCNL surgery. And the predictive performance of the B.T.C.H. nephrolithometry score surpassed that of other existing scoring systems, and it also effectively reflects the complexity of the PCNL procedure.

Stone burden is undeniably a key factor influencing the complexity and success rate of PCNL procedures. Previous studies have confirmed that stone burden significantly impacts the postoperative stone-free rate (SFR) of PCNL (11, 12). As stone burden increases, operative time extends, and surgical difficulty intensifies, particularly in cases of staghorn calculi, which often necessitate multi-tract PCNL or multi-stage procedures to achieve complete clearance. Consequently, the existing PCNL scoring systems incorporate stone burden as a critical evaluation criterion to predict surgical difficulty and SFR (13).

Anatomical variations of the renal pelvicalyceal system are critical factors influencing the outcomes of PCNL, yet they are often overlooked and not accounted for in existing PCNL scoring systems. Numerous studies have highlighted that the renal pelvicalyceal system exhibit significant individual variability, much like fingerprints (14). In our study, we primarily categorized the renal pelvis into two types: single pelvis and divided renal pelvis. It is intuitive that for patients with a divided pelvis, a single access tract may not reach stones located in the other branch, significantly increasing the complexity of achieving complete stone clearance in PCNL, and additional tracts were often necessary. Therefore, when scoring, the divided pelvis is rated as 3 points. Our findings also demonstrated that the prevalence of divided pelvis was significantly higher in the non-stone-free group compared to the stone-free group. While many recent studies have proposed various classifications for the renal pelvis and calyceal system (15, 16), and cases with anatomical features such as small renal pelvises and long, narrow infundibula significantly increase the difficulty of PCNL surgery. it remains unclear which classification or anatomical features could distinguish the complexity of PCNL procedures, which need further study to explore.

The distribution of stones significantly affects the stone-free outcomes of PCNL procedures (17). When the distribution of stone is more scattered, a single access tract may be insufficient to reach all stones, necessitating the use of multiple tracts for cleaning all stone. Additionally, if stones are present in calyces parallel to the punctured calyx, multiple tracts are often required to achieve complete stone clearance. Our scoring system employs the number of involved calyces as an indicator of stone distribution, with a higher number of involved calyces indicating a more scattered distribution. Similar to the S-ReSC (5), which incorporates stone distribution as part of its evaluation criteria by scoring the involvement of regions of the collecting system. Previous studies have also validated that the number of calyces involved by stones is a crucial predictor of PCNL outcomes (18).

The ideal cases for ultrasound-guided percutaneous nephrolithotomy (PCNL) are non-staghorn stones accompanied by mild hydronephrosis, as such cases can significantly reduce the difficulty of the puncture (19). In contrast, cases without hydronephrosis often present with complete staghorn stones, which increases the difficulty of puncture. Artificial hydronephrosis can significantly reduce the difficulty of ultrasound-guided puncture. However, for cases with local hydronephrosis caused by the obstruction of the renal calyx infundibulum and cases without hydronephrosis caused by complete staghorn calculi, artificial hydronephrosis cannot change the hydronephrosis situation of the punctured calyx. Studies have confirmed that the absence of hydronephrosis is a risk factor for residual stones postoperatively (20). Therefore, the degree of hydronephrosis significantly impacts the difficulty of PCNL, particularly in terms of puncture complexity. For cases with localized hydronephrosis, caused by obstruction of the renal calyx infundibulum, the difficulty of stone clearance is increased.

This study has several limitations. Firstly, the scoring criteria of B.T.C.H. nephrolithometry score were based on CTU evaluation, which cannot be performed in patients with impaired renal function, introducing a selection bias. Secondly, the B.T.C.H. nephrolithometry score has been validated only at our center, and further external validation with large samples from other centers is needed to evaluate its predictive performance.

## 5 Conclusion

B.T.C.H. nephrolithometry score is a suggested novel scoring system for ultrasound-guided percutaneous nephrolithotomy, which had superior prediction of stone-free rate and positive correlation with operative time, the number of tracts, and postoperative CDC scores.

## Data Availability

The data analyzed in this study is subject to the following licenses/restrictions: the datasets used and/or analyzed during the current study are available from the corresponding author on reasonable request. Requests to access these datasets should be directed to lijianxing2015@163.com.
